# Near infrared photoimmunotherapy with an anti-mesothelin antibody

**DOI:** 10.18632/oncotarget.8025

**Published:** 2016-03-10

**Authors:** Tadanobu Nagaya, Yuko Nakamura, Kazuhide Sato, Yi-Fan Zhang, Min Ni, Peter L. Choyke, Mitchell Ho, Hisataka Kobayashi

**Affiliations:** ^1^ Molecular Imaging Program, Center for Cancer Research, National Cancer Institute, National Institutes of Health, Bethesda, Maryland, 20892, USA; ^2^ Laboratory of Molecular Biology, Center for Cancer Research, National Cancer Institute, National Institutes of Health, Bethesda, Maryland, 20892, USA

**Keywords:** near infrared photoimmunotherapy, mesothelin, hYP218, humanized monoclonal antibodies, molecular imaging

## Abstract

Near Infrared-Photoimmunotherapy (NIR-PIT) is a new, highly selective tumor treatment that employs an antibody-photon absorber conjugate (APC). When the APC attaches to its target cell and is exposed to NIR light, highly selective cell killing is observed. NIR-PIT has been demonstrated with a limited number of antibodies. Mesothelin is overexpressed in several malignancies and is emerging as a therapeutic target. A recently humanized antibody (hYP218) has been generated against mesothelin that demonstrates high affinity binding. Here, we describe the efficacy of NIR-PIT, using hYP218 as the antibody within the APC to target a mesothelin expressing A431/H9 cell. The hYP218 antibody was conjugated to a photo-absorber, IR700 and incubated with the cells. The hYP218-IR700 showed specific binding to cells and cell-specific killing was observed *in vitro*. After implanting A431/H9 cells in an athymic nude mouse, tumor-bearing mice were treated with the following regimen of NIR-PIT; 100 μg of hYP218-IR700 i.v., NIR light was administered at 50 J/cm^2^ on day 1 after injection and 100 J/cm^2^ of light on day 2 after injection. The hYP218-IR700 showed high tumor accumulation and a high tumor-background ratio (TBR). Tumor growth was significantly inhibited by NIR-PIT treatment compared with the other control groups (*p* < 0.001), and significantly prolonged survival (*p* < 0.0001 vs other groups). Thus, the new anti-mesothelin antibody, hYP218, is suitable as an antibody-drug conjugate for NIR-PIT. Furthermore, NIR-PIT with hYP218-IR700 is a promising candidate for the treatment of mesothelin-expressing tumors that could be readily translated to humans.

## INTRODUCTION

Mesothelin is a cell surface glycoprotein and tumor differentiation antigen expressed in several aggressive tumors namely, mesothelioma [1], pancreatic adenocarcinoma [2–4], ovarian cancer [4, 5] and lung adenocarcinoma [4, 6]. Mesothelin can be used as a systemic diagnostic biomarker because it is shed from the cell surface in biofluids such as serum, plasma, and pleural effusions [7–9]. Additionally, its identification as a cancer cell antigen has helped mesothelin emerge as a target for antibody-based therapies [10]. A number of anti-mesothelin monoclonal antibodies (mAb) including SS1P, anti-mesothelin immunotoxin, and MORAb-009 (amatuximab), a chimeric anti-mesothelin mAb, have been developed and evaluated in clinical studies [11, 12]. SS1P and MORAb-009 recognize an epitope within the N-terminal Region I (residues 296–390) of cell surface mesothelin [13]. Unfortunately, Region I also interacts with MUC16 (also known as CA125) which may interfere with the binding and function of the antibody. This may compromise the efficacy of these antibodies. Recently, a humanized non-Region I antibody (hYP218) with high binding affinity for the C-terminus (residues 487–581) of mesothelin has been reported. There have been encouraging reports on its mesothelin-specific cytotoxicity [14].

Near infrared photoimmunotherapy (NIR-PIT) is a newly developed cancer treatment that employs a targeted monoclonal antibody-photon absorber conjugate (APC) [15]. The photon absorber, IRDye700DX (IR700, silica-phthalocyanine dye), is a highly hydrophilic dye which sets it apart from the hydrophobic dyes normally used in photodynamic therapy (PDT). NIR-PIT has been shown to be effective with a variety of different antibodies, but has not been tested with anti-mesothelin antibodies [16–20].

In this study, we investigated hYP218-IR700 as a candidate APC to be used in NIR-PIT. The mesothelin expressing A431/H9 cell line was used to study *in vitro* tumor binding, *in vivo* tumor accumulation and intratumoral distribution. NIR-PIT was performed using hYP218-IR700 *in vitro* and in a tumor-bearing mouse model *in vivo*.

## RESULTS

### *In vitro* characterization of A431/H9 cell

**Figure 1:**
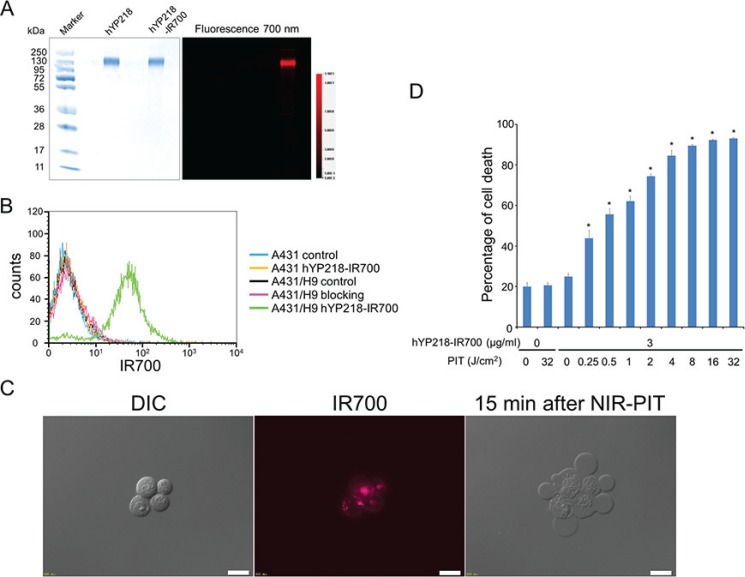
Confirmation of mesothelin expression as a target for NIR-PIT in A431/H9 cells, and evaluation of *in vitro* NIR-PIT (**A**) Validation of hYP218-IR700 by SDS-PAGE (left: Colloidal Blue staining, right: fluorescence). Diluted hYP218 was used as a control. (**B**) Expression of mesothelin in A431 and A431/H9 cells was examined with FACS. After 6 hours of hYP218-IR700 incubation, A431/H9 cells showed high fluorescence signal. (**C**) Differential interference contrast (DIC) and fluorescence microscopy images of A431/H9 cells after incubation with hYP218-IR700 for 6 h. High fluorescence intensities were shown in A431/H9 cells. Necrotic cell death was observed upon excitation with NIR light (after 15min). Scale bars = 20 μm. (**D**) Membrane damage of cells induced by NIR-PIT was measured with the dead cell count using PI staining, which increased in a light dose dependent manner (*n* = 5, *p < 0.001, vs. untreated control, by Student's *t* test).

As defined by SDS-PAGE, the band of hYP218-IR700 was almost the same molecular weight as the non-conjugate control, and fluorescence intensity was identical (Figure 1A). After a 6 h incubation with hYP218-IR700, A431/H9 cells showed a high fluorescence signal, which was confirmed with flow cytometry and fluorescence microscopy (Figure 1B and 1C).

On the other hand, mesothelin negative A431 cells did not show an increase in fluorescence signal after hYP218-IR700 incubation. Additionally, this increase in fluorescence signal was blocked by adding excess hYP218, indicating that hYP218-IR700 specifically binds to the mesothelin on A431/H9 cells.

### *In vitro* NIR-PIT

Immediately after exposure, NIR light induced cellular swelling, bleb formation, and rupture of vesicles. All of these changes are representative of necrotic cell death (Supplementary Video). Most of these morphologic changes were observed within 15 min of light exposure (Figure 1C), indicating rapid induction of necrotic cell death. Based on incorporation of PI, percentage of cell death increased in a light dose dependent manner (Figure 1D). Over 80% of A431/H9 cells died when exposed to 4 J/cm^2^ of NIR light. There was no significant cytotoxicity associated with NIR light alone in the absence of APC and with APC alone without NIR light.

### *In vivo* fluorescence imaging studies

**Figure 2:**
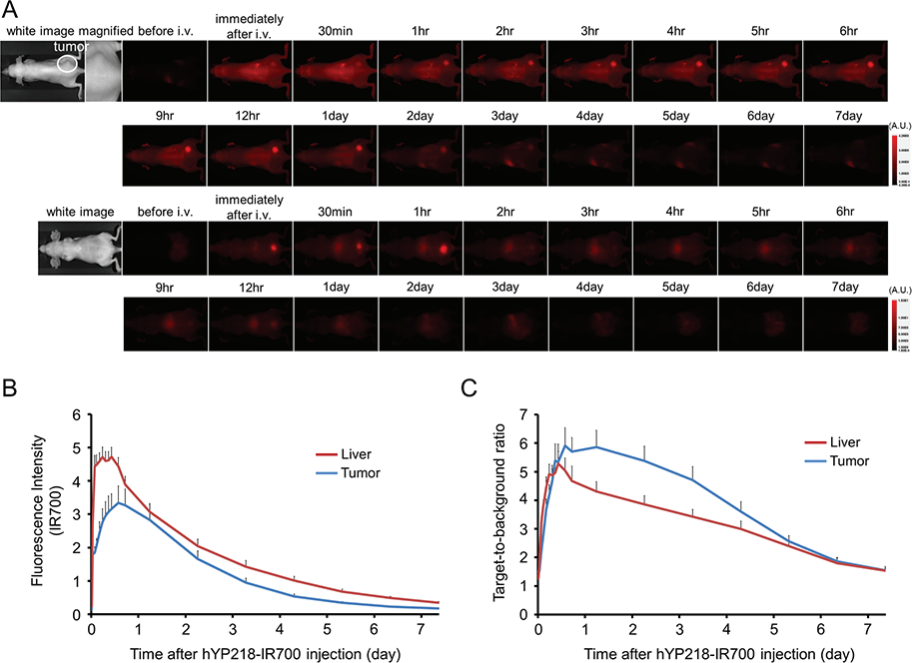
*In vivo* fluorescence imaging of A431/H9 tumor (**A**) *In vivo* hYP218-IR700 fluorescence real-time imaging of tumor bearing mice (right dorsum). The tumor showed high fluorescence intensity after injection and the intensity gradually decreased over days. Most of the excess agent was excreted into the urine immediately after injection. (**B**) Quantitative analysis of IR700 intensities in tumor and liver (*n* = 10). The IR700 fluorescence intensity of tumor and liver shows high intensities within 1 day after APC injection but this decreases gradually over days. (**C**) Quantitative analysis of TBR in tumors and livers (*n* = 10). TBR of tumor is high within 1 day after APC injection. However, TBR of liver decreased starting 6 hours after APC injection.

The fluorescence intensity and TBR of hYP218-IR700 in A431/H9 tumors decreased gradually over days (Figure 2). Similarly, the fluorescence intensity and TBR of hYP218-IR700 in the liver decreased gradually over days (Figure 2). To obtain the maximal therapeutic effect the fluorescence of the APC should be high in the tumor and low in the background. Tumors still showed high fluorescence intensity one day after APC injection, while fluorescence signal of background including liver decreased beginning 6 hours after APC injection. Thus, we used one day after APC injection to get the maximal difference between tumor and background normal tissue.

### *In vivo* NIR-PIT

**Figure 3:**
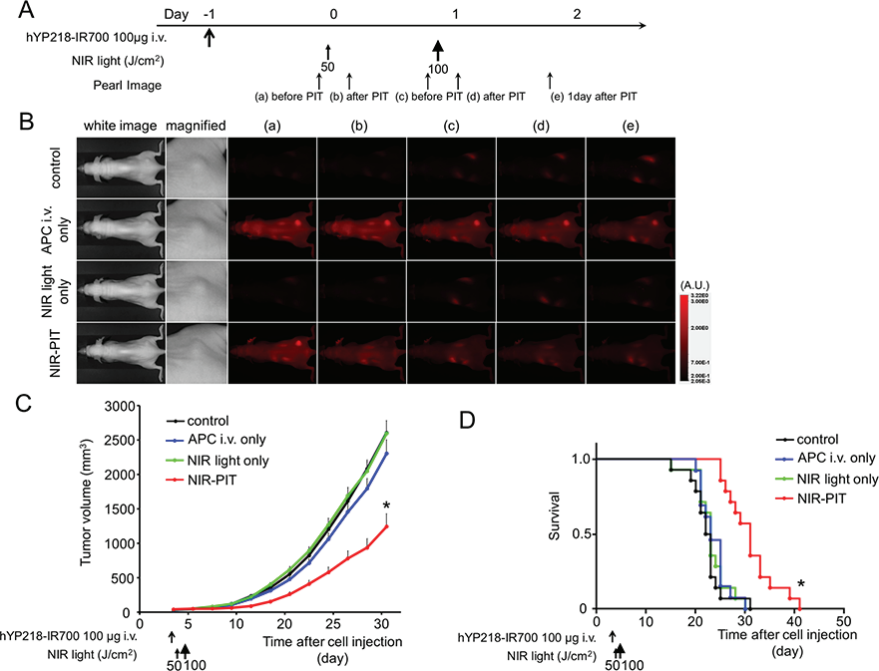
*In vivo* effect of NIR-PIT for A431/H9 tumor (**A**) NIR-PIT regimen. Fluorescence images were obtained at each time point as indicated. (**B**) *In vivo* fluorescence real-time imaging of tumor bearing mice in response to NIR-PIT. The tumor treated by NIR-PIT showed decreasing IR700 fluorescence after NIR-PIT. (**C**) Tumor growth was significantly inhibited in the NIR-PIT treatment groups (*n* ≧ 10, *p < 0.001 vs other groups, Bonferroni's test with ANOVA). (**D**) Significantly prolonged survival was observed in the NIR-PIT treatment group (*n* ≧ 10, *p < 0.0001 vs other groups, by Log-rank test).

The treatment and imaging regimen is shown in Figure 3A. One day after injection of hYP218-IR700, tumors showed higher fluorescence intensity than the tumors with no APC. After exposure to 50 J/cm^2^ of NIR light, IR700 fluorescence signal decreased due to dying cells and partial photo-bleaching. The IR700 fluorescence did not change for up to two days in tumors receiving hYP218-IR700 but not NIR light (Figure 3B). Tumor growth was significantly inhibited in the NIR-PIT treatment group compared with the other groups (*p* < 0.001) (Figure 3C). Additionally, significantly prolonged survival was achieved in the NIR-PIT group (*p* < 0.0001 vs other groups) (Figure 3D). No significant therapeutic effect was observed in the control groups including those receiving APC *i.v.* only or in mice receiving the NIR light only. There was no skin necrosis or toxicity attributable to the APC in any group.

### Histological analysis

**Figure 4:**
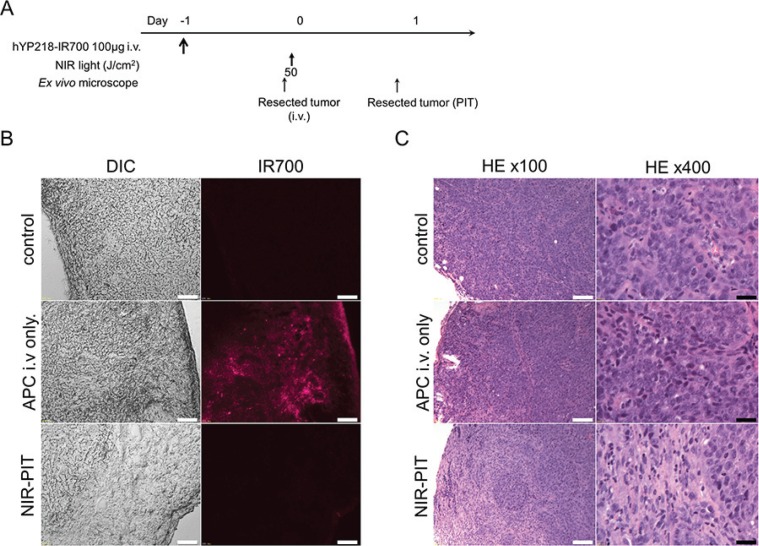
*In vivo* histological fluorescence distribution and histological NIR-PIT effect (**A**) The regimen of NIR-PIT. (**B**) Differential interference contrast (DIC) and fluorescence microscopy images of A431/H9 tumor xenografts. High fluorescence intensity is shown in A431/H9 cells 24 h after injection of hYP218, but the fluorescence disappears 24 h after NIR-PIT. Scale bars = 100 μm. (**C**) Resected tumor stained with Hematoxylin and Eosin. A few scattered clusters of damaged tumor cells are seen within a background of diffuse cellular necrosis and micro-hemorrhage after NIR-PIT, while no obvious damage was observed after hYP218-IR700 alone with NIR light. White scale bars = 100 μm. Black scale bars = 20 μm.

In frozen histologic specimen, high fluorescence intensity was shown in A431/H9 cells 24 h after hYP218-IR700 injection compared with that in control A431 cells. On the other hand, the majority of fluorescence signal in A431/H9 cells disappeared 24 h after NIR-PIT (Figure 4B). In the H & E stained histologic specimen, NIR-PIT treated A431/H9 tumors showed diffuse necrosis and micro-hemorrhage with scattered clusters of remaining damaged tumor cells. However, no damage was observed in A431/H9 tumors 24 h after hYP218-IR700 injection without NIR light exposure (Figure 4C).

## DISCUSSION

Mesothelin has been reported to be expressed in various tumors such as malignant mesotheliomas and pancreatic ductal adenocarcinoma among others [1–5]. Malignant mesothelioma is an aggressive disease with poor prognosis that exhibits high expression of mesothelin [21]. In patients with unresectable disease, combination chemotherapy is the most effective therapy, but median overall survival is only 12.1 months [22]. Advanced pancreatic cancer, which also is a mesothelin expresser, has a median survival of only 11.1 months even after combination chemotherapy [22, 23]. Thus, the fact that mesothelin is expressed on some aggressive cancers and that there is limited normal expression of mesothelin gives impetus to the development of antibodies targeting the mesothelin antigen [10].

SS1P and MORAb-009 are anti-mesothelin antibodies that recognize an epitope within the N-terminal Region I (296–390) of cell surface mesothelin [13]. However, Region I is a common site for the binding of MUC16 and thus the antibody can be competitively inhibited. Humanized YP218 is a fully humanized non-Region I anti-mesothelin, that does not compete with anti-mesothelin Region I antibodies [14]. The conjugated hYP218-IR700, achieved adequate tumor TBRs as shown in Figure 2, indicating that it may be practical for clinical application during surgical or endoscopic procedures because of its high TBR on the mesothelin expressing tumors. Efficient binding and distribution of the antibody in tumors are important for APCs such as the one described here and antibody-toxin or antibody-drug conjugates. Additionally, the drugs and toxins must be internalized after cell binding in order to be effective. Our results showed that hYP218 bound to mesothelin specifically and was internalized homogenously within 6 hours of incubation in mesothelin expressing cancer cells. These results suggest that hYP218 has favorable characteristics for an antibody-drug conjugate.

The conjugate hYP218-IR700 proved to be an effective agent for treating a mesothelin expressing tumor model with NIR-PIT. NIR-PIT with hYP218-IR700 led to rapid cell death *in vitro* and significant tumor growth reduction and survival improvement *in vivo*. Thus, hYP218-IR700 could be an effective platform for NIR-PIT in mesothelin expressing tumors.

The delivery of various nano-sized or macromolecular drugs to tumor beds increases up to a 24-fold within 8 hours after NIR-PIT compared with that into non-treated tumor beds. NIR-PIT has been shown to results in super enhanced permeability and retention (SUPR) effects [24–26]. When intravenously injected, APCs are delivered via tumor vasculature into tumor beds. Thus, APCs can bind to cancer cells in the perivascular space at the highest concentration. When exposed NIR light, the cancer cells in the perivascular space are killed leading to a dramatic increase in vascular permeability. After the first NIR-PIT, circulating APCs perfuse deep into treated tumor beds and bind surviving cancer cells. Therefore, the second exposure to NIR light can further enhance therapeutic effects of NIR-PIT [27]. Thus, we chose the current therapeutic regimen that employed a single injection of APCs and two serial NIR light exposures.

An obvious limitation of NIR-PIT for mesothelin expressing tumor is the inability to deliver NIR light to the tumor. Skin, fat and other organs, will absorb NIR light before it reaches the tumor. There are several potential solutions to this problem. For instance, NIR light could be delivered to a pleural or peritoneal mesothelioma or pancreatic tumor while the tissues are still exposed after surgical resection, thus treating residual tumor. Alternatively, light could be administered endoscopically, via a bronchoscope in the case of pleural mesothelioma or via a gastrointestinal endoscope in the case of pancreatic cancer. A third possibility is to introduce light fibers interstitially into tumors using needle trocars, although this might be of limited value in mesothelioma. Such procedures have been proposed in the past with PDT, however, we believe that NIR-PIT would be much more effective with lower toxicity than PDT [28, 29]. An alternative light based cell selective cancer therapy is fluorescence guided surgery which utilized the telomerase-dependent expression of green fluorescent protein (GFP). This gene is transfected using adenovirus and allows for curative resection of various tumors [30–32]. However, this therapy requires virus mediated transfection of the GFP gene into cancer cells. This might complicate the approval by regulatory agencies when compared to the antibody based NIR-PIT. Therefore, NIR-PIT would be technically easier for transitioning into the clinical practice.

This study has several limitations. The cell line used demonstrated very high expression of mesothelin. It is known that there is variable expression in tumors known to express mesolthelin and some tumors may express insufficient amounts of antigen for effective NIR-PIT. This study was also done in an immune incompetent mouse model. As the immune effects of NIR-PIT are currently unknown, it is difficult to know whether this will be of benefit to a patient, although it is anticipated that it will augment the therapeutic effect. Another caveat in this study is the use of subcutaneously xenografted human tumors in athymic mice does not fully represent human cancers. Superior tumor models including surgically implanted patient derived orthotopic tumor models, can simulate cancers in patients better than xenografted tumor models [33, 34]. These orthotopic models required highly trained surgical skills for proper implanting of the tumor and are much more invasive than s.c. injections. Therefore, in this proof-of-principle study of NIR-PIT targeting mesothelin, we chose a simple subcutaneous xenograft tumor model. Finally, we only performed one injection of the APC with two exposures of light. Clearly, repeated dosing of the APC with repeated light exposure is likely to increase effectiveness. For instance, epidermal growth factor receptor (EGFR)-targeted NIR-PIT in an appropriate model with a repeated regimen of APC and light dosing has been reported to improve therapeutic effect [27, 35]. However, access to this new antibody is still limited and thus, only single injections of the conjugate were feasible at the time of this experiment.

## CONCLUSIONS

The fully humanized non-Region I anti-mesothelin antibody, hYP218, demonstrated adequate accumulation and internalization in mesothelin expressing cancer cells. The conjugated, hYP218-IR700 and NIR-PIT can be an effective treatment of a mesothelin expressing animal tumor model. Impressive, immediate responses were seen after only a single injection of the conjugate and two light exposures. It would be desirable to extend these studies to multiple doses of the APC and light, however, this awaits improved access to the antibody. If successful, NIR-PIT in mesothelin expressing, unresectable tumors such as mesothelioma and pancreatic cancer, could have a meaningful impact on the survival of such patients.

## MATERIALS AND METHODS

### Reagents

Water soluble, silica-phthalocyanine derivative, IRDye 700DX NHS ester was obtained from LI-COR Biosciences (Lincoln, NE, USA). All other chemicals were of reagent grade.

### Production of the hYP218 humanized antibody

#### Generation of a stable cell line

A high-affinity anti-mesothelin rabbit Fv was humanized (named hYP218) in the Laboratory of Molecular Biology, NCI as previously described [14]. The VH and VL sequences of hYP218 were subcloned into the pDR12 vector (kindly provided by Dennis Burton, Scripps Institute, La Jolla, CA). The resulting plasmid (pMH227) was transfected into CHO-S cells (Invitrogen, Carlsbad, CA) to make a stable line expressing hYP218 antibody (IgG1 and kappa light chain) for large scale production. The stable line was generated following a modified laboratory protocol described previously [36]. Briefly, the CHO-S cells were cultured in the F-12K medium supplemented with 10% FBS and 1% penicillin/streptomycin, and transfected with pMH227 using polyethylenimine (PEI 25 Kd linear, PolySciences, Warrington, PA). Transfected cells were grown for 3 days, then collected and resuspended in a selection medium containing the glutamine synthetase inhibitor, L-methionine sulfoximime (MSX, Sigma-Aldrich, St. Louis MO) in the absence of glutamine [Glasgow's Modified Eagle's Medium (GMEM) supplemented with 10% dialyzed FBS (Hyclone, Logan, UT), GS supplement, and 25 μM MSX]. Cells were seeded into 96 well plates at 10^5^ cells/well, and cultured for 1 month. The wells with stable colonies were further subcloned and screened for high antibody expression.

#### Screen for high antibody expression

A sandwich ELISA was performed to check the antibody titer in culture supernatant. Clones showing the highest production levels were expanded and transferred to suspension expression medium [SFM4CHO without glutamine (Hyclone)] supplemented with 25 μM MSX at 0.3−1 × 10^6^ cells/ml. The hYP218 antibody protein was purified from the culture supernatant on a Protein A column (GE Healthcare Life Sciences, Pittsburgh PA) using an ÄKTA Explorer system (GE Healthcare, Life Sciences) as previously described [37].

### Synthesis of IR700-conjugated hYP218

Conjugation of dyes with monoclonal antibody was performed according to previous report [15]. In brief, hYP218 (1.0 mg, 13.3 nmol) was incubated with IR700 NHS ester (130.3 μg, 66.7 nmol) in 0.1 M Na_2_HPO_4_ (pH 8.6) at room temperature for 1 h. The mixture was purified with a Sephadex G25 column (PD-10; GE Healthcare, Piscataway, NJ, USA). The protein concentration was determined with Coomassie Plus protein assay kit (Thermo Fisher Scientific Inc, Rockford, IL, USA) by measuring the absorption at 595 nm with UV-Vis (8453 Value System; Agilent Technologies, Santa Clara, CA, USA). The concentration of IR700 was measured by absorption at 689 nm to confirm the number of fluorophore molecules per mAb. The synthesis was controlled so that an average of two IR700 molecules was bound to a single antibody. As a quality control for the conjugate, we performed sodium dodecyl sulfate-polyacrylamide gel electrophoresis (SDS-PAGE). The Conjugate was separated by SDS-PAGE with a 4–20% gradient polyacrylamide gel (Life technologies, Gaithersburg, MD). A standard marker (Crystalgen Inc., Commack, NY) was used as a protein molecular weight marker. After electrophoresis at 80 V for 2.5 h, the gel was imaged with a Pearl Imager (LI-COR Biosciences, Lincoln, Nebraska, USA) using a 700 nm fluorescence channel. We used diluted hYP218 as non-conjugated control. The gel was stained with Colloidal Blue staining to determine the molecular weight of conjugate.

### Cell culture

A431 (epidermal carcinoma) and A431/H9, a cell line stably expressing human mesothelin [38], were used in this study. Cells were grown in RPMI 1640 (Life Technologies, Gaithersburg, MD, USA) supplemented with 10% fetal bovine serum and 1% penicillin/streptomycin (Life Technologies, Carlsbad, CA) in tissue culture flasks in a humidified incubator at 37^°^C in an atmosphere of 95% air and 5% carbon dioxide.

### Flow cytometry

To verify *in vitro* hYP218-IR700 binding, fluorescence from cells after incubation with APC was measured using a flow cytometer (FACS Calibur, BD BioSciences, San Jose, CA, USA) and CellQuest software (BD BioSciences). A431 and A431/H9 cells (2 × 10^5^) were seeded into 12 well plates and incubated for 24 h. Medium was replaced with fresh culture medium containing 3 μg/ml of hYP218-IR700 and incubated for 6 h at 37^°^C. To validate the specific binding of the conjugated antibody, excess antibody (30 μg) was used to block 3 μg of APCs.

### Fluorescence microscopy

To detect the antigen specific localization and effect of NIR-PIT, fluorescence microscopy was performed (BX61; Olympus America, Inc., Melville, NY, USA). Ten thousand cells were seeded on cover glass bottomed dishes and incubated for 24 h. Humanized YP218-IR700 was then added to the culture medium at 3 μg/ml and incubated for 6 h at 37^°^C. After incubation, the cells were washed with phosphate buffered saline (PBS). The filter set to detect IR700 consisted of a 590–650 nm excitation filter, a 665–740 nm band pass emission filter. Transmitted light differential interference contrast (DIC) images were also acquired.

### *In vitro* NIR-PIT

The cytotoxic effects of NIR-PIT with hYP218-IR700 were determined by flow cytometric Propidium Iodide (PI) (Life Technologies, Carlsbad, CA) staining, which can detect compromised cell membranes. Two hundred thousand cells were seeded into 12 well plates and incubated for 24 h. Medium was replaced with fresh culture medium containing 3 μg/ml of hYP218-IR700 and incubated for 6 h at 37^°^C. After washing with PBS, PBS was added and cells were irradiated with a red light-emitting diode (LED) which emits light at 670-710nm wavelength (L690-66-60; Marubeni America Co., Santa Clara, CA, USA) at a power density of 50 mW/cm^2^ as measured with an optical power meter (PM 100, Thorlabs, Newton, NJ, USA). Cells were scratched 1 h after treatment. Then PI was added in the cell suspension (final 2 μg/ml) and incubated at room temperature for 30 min, followed by flow cytometry. Each value represents mean ± standard error of the mean (s.e.m.) of five experiments.

### Animal and tumor models

All *in vivo* procedures were conducted in compliance with the Guide for the Care and Use of Laboratory Animal Resources (1996), US National Research Council, and approved by the local Animal Care and Use Committee. Six to eight week old female homozygote athymic nude mice were purchased from Charles River (NCI-Frederick, Frederick, MD). During the procedure, mice were anesthetized with isoflurane. In order to determine tumor volume, the greatest longitudinal diameter (length) and the greatest transverse diameter (width) were measured with an external caliper. Tumor volumes were based on caliper measurements and were calculated using the following formula; tumor volume = length × width^2^ × 0.5. Body weight was also measured. Mice were monitored daily for their general health and tumor volumes were measured three times a week until the tumor volume reached 2000 mm^3^, where upon the mice were euthanized with inhalation of carbon dioxide gas.

### *In vivo* fluorescence imaging studies

A431/H9 cells (2 × 10^6^) were injected subcutaneously in the right dorsum of the mice. Tumors were studied after they reached volumes of approximately 50 mm^3^. Serial ventral and dorsal fluorescence images of IR700 were obtained with a Pearl Imager using a 700 nm fluorescence channel before and 0, ½, 1, 2, 3, 4, 5, 6, 9, 12, 24, 48, 72, 96, 120, 144, and 168 hours after i.v. injection of 100 μg of hYP218-IR700 via the tail vein. Pearl Cam Software (LI-COR Biosciences, Lincoln, NE) was used for analyzing fluorescence intensities. Region of interests (ROIs) were placed on the tumor and liver. ROIs were also placed in the adjacent non-tumor region as background (left dorsum and lower abdomen). Average fluorescence intensity of each ROI was calculated. Target-to-background ratio (TBR) (fluorescence intensities of target/fluorescence intensities of background) were also calculated (*n* = 10).

### *In vivo* NIR-PIT

A431/H9 cells (2 × 10^6^) were injected subcutaneously in the right dorsum of the mice. Tumors were studied after they reached volumes of approximately 50 mm^3^. To examine the therapeutic effect of *in vivo* NIR-PIT on A431/H9 cells, tumor bearing mice were randomized into 4 groups of at least 10 animals per group for the following treatments: (1) no treatment (control); (2) 100 μg of hYP218-IR700 i.v., no NIR light exposure (APC i.v. only); (3) NIR light exposure only, NIR light was administered at 50 J/cm^2^ on day 1 and 100 J/cm^2^ on day 2 (NIR light only); (4) 100 μg of hYP218-IR700 i.v., NIR light was administered at 50 J/cm^2^ on day 1 after injection and 100 J/cm^2^ on day 2 after injection (NIR-PIT). Serial fluorescence images, as well as white light images, were obtained before and after each NIR light exposure (day 1 and day 2) using a Pearl Imager with a 700 nm fluorescence channel.

### Histological analysis

To detect the antigen specific micro-distribution in the tumor, fluorescence microscopy was performed. Tumor xenografts were excised from mice without treatment, 24 h after injection of hYP218-IR700 (APC i.v. only) and 24 h after NIR-PIT. Extracted tumors were frozen with OCT compound (SAKURA Finetek Japan Co., Tokyo, Japan) and frozen sections (10 μm thick) were prepared. Fluorescence microscopy was performed using the BX61 microscope with the following filters; excitation wavelength 590 to 650 nm, emission wavelength 665 to 740 nm long pass for IR700 fluorescence. DIC images were also acquired. To evaluate histological changes light microscopy study was also performed using Olympus BX61. Extracted tumors were also placed in 10% formalin and serial 10 μm slice sections were fixed on glass slide with Hematoxylin and Eosin (H & E) staining.

### Statistical analysis

Data are expressed as means ± s.e.m. from a minimum of five experiments, unless otherwise indicated. Statistical analyses were carried out using GraphPad Prism version 6 (GraphPad Software, La Jolla, CA, USA). For multiple comparisons, a one-way analysis of variance (ANOVA) followed by the Bonferroni's correction for multiple comparisons was used. The cumulative probability of survival based on volume (2000 mm^3^) were estimated in each group with a Kaplan-Meier survival curve analysis, and the results were compared with use of the log-rank test. Student's *t* test was used to compare the treatment effects with that of control. *P*-value of < 0.05 was considered statistically significant.

## SUPPLEMENTARY MATERIALS VIDEOS





## Abbreviations

APC: antibody-photon absorber conjugate
DIC: differential interference contrast
EGFR: epidermal growth factor receptor
GFP: green fluorescent protein
H & E: Hematoxylin and Eosin
IR700: IRDye700DX
LED: light-emitting diode
mAb: monoclonal antibodies
NIR: near-infrared
PI: propidium iodide
PBS: phosphate buffered saline
PIT: photoimmunotherapy
PDT: photodynamic therapy
ROI: regions of interest
SUPR: super enhanced permeability and retention
TBR: target-to-background ratio
