# Supplementary material for: Near infrared photoimmunotherapy with an anti-mesothelin antibody
Source: Oncotarget. 2016 Mar 10;7(17):23361–9. doi: 10.18632/oncotarget.8025 (PMC5029632; doi:10.18632/oncotarget.8025)
Supplement: Supplementary file 1 [file oncotarget-07-23361-s001.pdf]

Near infrared photoimmunotherapy with an anti-mesothelin antibody

Supplementary Materials

Supplementary Video: NIR-PIT effect for A431/H9 cells. Immediately after exposure to excitation light cellular swelling, bleb formation, and rupture of vesicles representing necrotic cell death were observed.
